# Towards Cell free Therapy of Premature Ovarian Insufficiency: Human Bone Marrow Mesenchymal Stem Cells Secretome Enhances Angiogenesis in Human Ovarian Microvascular Endothelial Cells

**DOI:** 10.24966/srdt-2060/100019

**Published:** 2019-11-06

**Authors:** Hang-Soo Park, Dalia Ashour, Amro Elsharoud, Rishi Man Chugh, Nahed Ismail, Abdeljabar EL Andaloussi, Ayman Al-Hendy

**Affiliations:** 1 Department of Surgery, University at Illinois at Chicago, Medical College, Chicago, USA; 2 Department of Pathology, University at Illinois at Chicago, Medical College, Chicago, USA

## Abstract

Primary Ovarian Insufficiency (POI) refers to an ovarian loss of function in women under the age of 40. Unfortunately, currently, there is no effective treatment available for POI-related infertility. Alternatives such as the use of egg donations are culturally and ethically unacceptable to many couples. Human Bone marrow-derived Mesenchymal Stem Cells (MSCs) are known for their ability to differentiate into other cell types, once primed by the organ microenvironment. Importantly MSCs produce a vast array of bioactive factors many of them have been shown to enhance neovascularization in various tissues. Recently, preliminary data from our ongoing clinical trial revealed encouraging preliminary data after autologous MSC engraftment into the ovaries of 2 POI patients with durable elevation in serum estrogen levels and increase in size of treated ovaries sustained up to one-year post cell therapy.

In this study, we investigated the action of the mechanisms of MSCs treatment on a POI ovary. We designed an in vitro study using MSC secretome and Human Ovarian Endothelial Cells (HOVECs) to understand the molecular mechanisms by which MSC mediates their angiogenic properties and regenerative effects. Human primary HOVECs were treatment with MSC secretome and examined by FACS for the expression of angiogenesis markers such as Endoglin, Tie-2, and VEGF. The formation of vessels was evaluated by using a 3D Matrigel tubulogenesis assay.

We observed that the expression of proliferation marker Ki67 was significantly increased under treatment with MSC secretome in HOVEC cells (P4). MSCs secretome treatment also induced significantly higher expression of several angiogenic markers such as VEGFR2, Tie2/Tek, VE-Cadherin, Endoglin, and VEGF compared to matched control (P4). Furthermore, MSC secretome significantly increased the number of branching points in tubulogenesis assay (P4). Our study suggests that MSC secretome likely contains bioactive factors that can enhance ovarian angiogenesis. Further characterization of these factors can lead to novel therapeutic options for women with premature ovarian insufficiency and other related causes of female infertility.

## 2. Introduction

Premature Ovarian Failure (POF) or Primary Ovarian Insufficiency (POI) is a condition where the loss of normal ovarian function happens before the age of 40. Patients with POI usually present with amenorrhea and infertility secondary to anovulation. The condition is marked with elevated serum levels of Follicle-Stimulating Hormone (FSH), decreased Anti-Mullerian Hormone (AMH) and low serum levels of estrogen [1–3]. Millions of women are diagnosed with cancer globally every year and many of these women are in reproductive and pre-reproductive age. As chemotherapy continues to be the treatment of choice for cancer, many of these women develop POI as a result of the genotoxic effects of various chemotherapeutic agents [4–6]. Although chemotherapy for the treatment of cancer in young women has dramatically improved their survival, Premature Ovarian Insufficiency (POI) is a common, long-term consequence due to chemotherapy-induced ovarian damage [4,5].

Many previous studies suggested various approaches to find out effective treatment for POI patients. According to recent studies and our previous work, one of the promising approaches to treat POI is cell therapy using MSC [6–8]. Human Mesenchymal Stem Cells (MSC) are one of the multipotent adult stem cells which can be isolated from mesodermal originated tissues such as bone marrow, dental pulp, and adipose tissue. MSC has been reported for its various therapeutic effects. MSC transplantation has been cited in more than 344 clinical trials [7]. The MSC is also well known as angiogenesis regulator. Many previous studies revealed that MSC can stimulate angiogenesis [9–12].

This angiogenic effect of MSC can be a protection against cell death induced by chemotherapy as a result of vascular damage. MSC is also well known as a regulator of inflammation. It has been reported that MSC secretome suppresses inflammatory response [13–15]. Another advantage of MSC is immune evasion. Many studies revealed that MSC can avoid immune reaction [16–19] and this gives MSC a great advantage in allogeneic transplantation.

Previously, we reported that human bone marrow-derived MSC transplantation in POI mice models can increase the size of ovary and reverse fertility [7]. However, it is still not clear how MSC can affect a POI ovary and the mechanisms of its action. We hypothesize that those abilities of MSC which described above can explain how MSC can revive the ovarian function.

In this study, we focused on angiogenesis, and we hypothesize that MSC can restore POI ovary function by stimulating vascular regeneration in the ovary.

To study ovarian angiogenesis under MSC treatment in vitro, we used human ovarian microvascular endothelial cells (HOVEC) and conditioned media from MSC (MSC CM). We treated MSC CM while culture HOVEC cells. Then compare angiogenesis marker expression and tube formation ability of HOVEC.

## 3. Material and Method

### 3.1. Cell culture (HOVEC, MSC)

Human ovarian microvascular endothelial cells (HOVEC) were purchased from ABM (ABM Inc., Richmond, BC, Canada). HOVEC cells were plated into recommended pre-coated cell culture flasks (ABM Inc., Richmond, BC, Canada) and cultured at standard cell culture condition at 5% CO_2_ and 37°C with endothelial culture media containing M199 medium (Gibco, Waltham, MA, USA) supplemented with 10% fetal bovine serum (FBS, Gibco, Waltham, MA, USA), and endothelial cell growth supplement from bovine pituitary (ECGS, Gibco, Waltham, MA, USA). Culture media were changed every 2 days. Cells were passaged with 0.5% trypsin-EDTA (Gibco, Waltham, MA, USA) solution when the culture reaches about 80 to 90% confluence in the culture flask. When the confluency reaches 90%, cells were split to the next passage.

Human bone marrow-derived mesenchymal stem cells (MSC) were purchased from Lonza (cat no: PT-2501, Switzerland) for this study which isolated from 24 years old healthy female donor. MSC were plated into cell culture flask with DMEM/F12 (Gibco, Waltham, MA, USA) based MSC culture media, containing, 10% FBS (Omega, Tarzana, CA, USA), and 1% of penicillin/streptomycin solution (Gibco, Waltham, MA, USA) in the final volume of media.

### 3.2. Collection and treatment with conditioned media of mesenchymal stem cells

When the MSC culture reaches about 80 to 90% in confluence, spent media from the flask were discarded and the cells were washed three times with PBS to complete removal of serum. Cells were cultured with DMEM/F12 media (Gibco, Waltham, MA, USA) without serum for 24 hours to collect the conditioned media. After 24 hours, MSC conditioned media (MSC CM) were collected from MSC flasks and centrifuge at 500g for 5 min at 40°C to remove any cell debris. MSC CM was aliquoted and stored at −80°C for further use. DMEM/F12 media (Gibco, Waltham, MA, USA) without serum and cells were also incubated from 24 hours in culture flask to be used as a negative control. HOVEC cells were treated for 24 hours with the MSC CM pre-diluted with endothelial medium without serum (regular media) in 1:1 ratio. After MSC CM treatment, HOVEC cells were collected for further analysis by flow cytometry and PCR.

### 3.3. Flow cytometry analysis

After the treatment with MSC CM and respective control, HOVEC cells were collected and analyzed for their proliferative enhancement and expression of angiogenesis marker. Following antibodies Ki 67, PE anti-human CD31, PE/ Cy7 anti-human CD144 (VE-Cadherin), PE anti-human CD309 (VEGF-R2), Alexa Fluor 647 anti-human CD202b (Tie2/Tec), PE anti-human CD105 (Endoglin) (BioLegend, San Diego, CA, USA) were used for analysis by FACS. In brief, after the treatment, cell pellets were harvested, fixed overnight then 50μl of cell suspension was incubated with 1ul of the antibody for 25 min at 4°C in 50 μl of PBS. For analysis 10 000 cells were analyzed on a FACScan (Becton Dickinson).

### 3.4. RT-PCR

RNA was extracted from control HOVEC and MSC CM treated HOVEC by TRIzol method. 500μL of TRIzol (Thermo, Waltham, MA, USA) was used for 200,000 HOVEC cells. The RNA was purified according to manufacturer’s protocol. After RNA purification, 500ng of RNA was used for cDNA conversion. Expression of angiogenesis markers is analyzed with RT2 profiler PCR array kit (QIAGEN, Hilden, Germany) and CFX96 PCR instrument (Bio-Rad, Hercules, CA, USA). Reaction volume per each well was 25μL, which containing 4ng of cDNA and 12.5μLof Universal SYBR green Supermix (Bio-Rad, Hercules, CA, USA). PCR was performed with cycle condition described in manufacturer’s protocol. Gene expression levels are analyzed by the delta-delta CT method.

### 3.5. Endothelial cell tube formation assay

Corning Matrigel Matrix (10 mg protein/ml) Growth Factor Reduced (GRF) (Corning Cat. No. 354230) was used for tube formation assay. 289μL of cold (4°C) Matrigel per well was added into chilled added into 24-well flat-bottom tissue culture plate (Scientific Inc.) and polymerized for 1 h at 37°C, 5% CO_2_incubator. HOVECs were trypsinized, separated into three different groups. Each group of cells centrifuged and cell pellets were resuspended in regular media without FBS, same media with FBS, and 1:1 mixture of FBS free regular media and MSC CM. Then 300 μl of the cell suspension was added onto each well of Matrigel coated 24-well culture plate and incubated at 37°C. After 18 h, and finally, cells forming tube structure, and analyzed by microscopy. Cells in five replicate fields of triplicate wells were digitally photographed with an EVOS microscope (Thermo, Waltham, MA, USA). The total tubular length was quantified by image analysis software (Image J). The total tubular length was calculated as the average of the total tubule length from three wells, five random fields per well.

### 3.6. Statistics

All statistical analyses were performed using IBM SPSS Statistics 25 (IBM, Armonk, NY, USA) assuming an overall significance level by P-value (P<0.05) in paired T-test.

## 4. Results

### 4.1. MSC CM enhances proliferation of HOVEC

**Figure 1:**
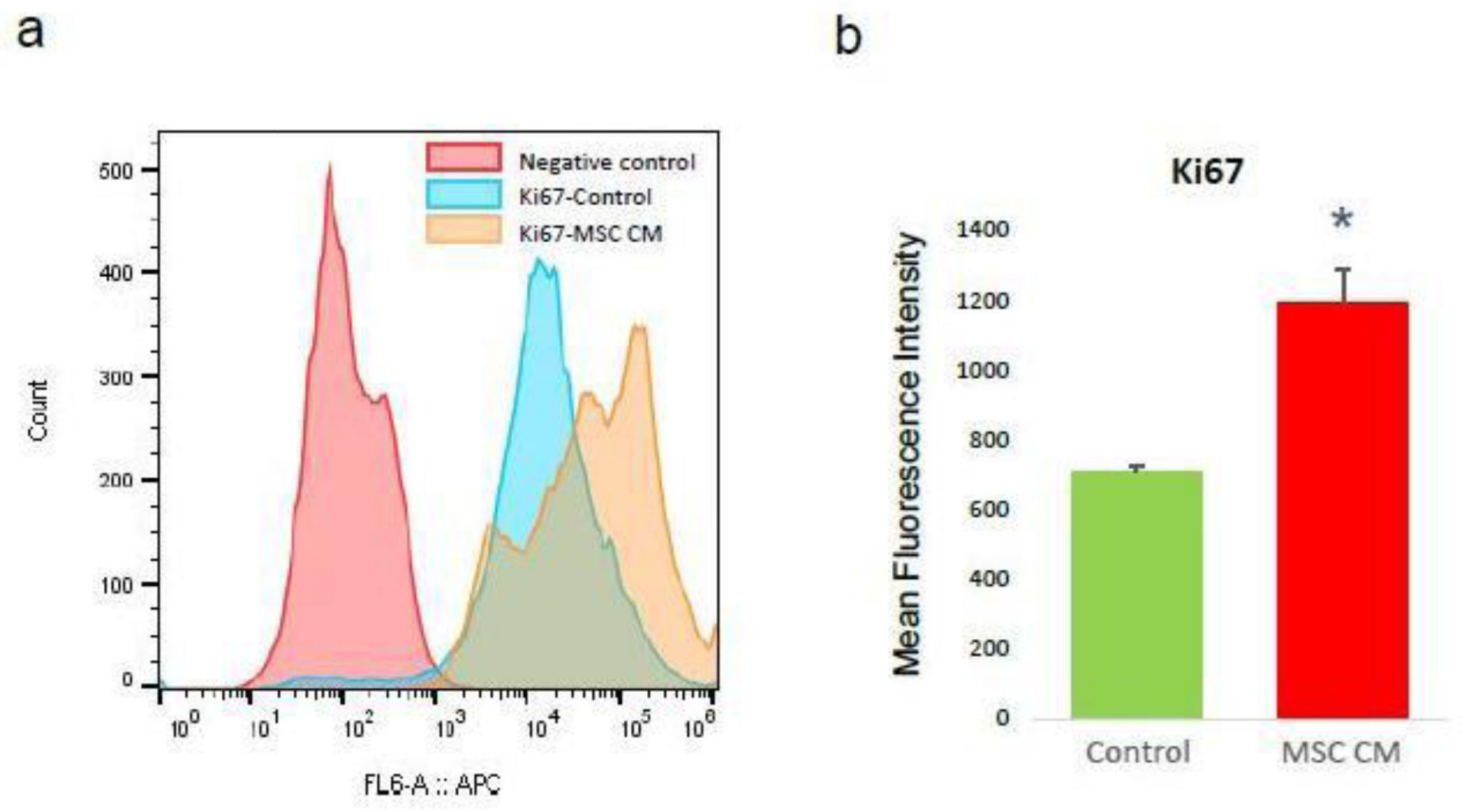
Proliferation analysis of HOVEC cells. HOVEC cells which were cultured with control media (control) and 50% MSC CM containing media (MSC CM) are stained with APC conjugated anti-Ki67 antibody and analyzed Ki67 positive population using FACS. (a) Histogram of Ki67 positive HOVEC cells. (b) Quantitative display for Mean Fluorescence Intensity of Ki67 in control and MSC CM treated HOVEC cells. (P<0.05).

We hypnotized that MSC can stimulate ovarian angiogenesis in POI ovary by enhancing proliferation of endothelial cells or stimulation of angiogenesis ability. As a first step, we analyzed the effect of MSC CM on the proliferation of endothelial cells. To analyze the effect of MSC CM, we divided HOVEC cells into two groups. The test group HOVEC cells was treated with MSC CM, and the control group HOVEC cells was treated with control media. After treatment for 24h, we harvested the cells from each group and analyzed cell proliferation marker Ki67 using FACS. We quantified the protein expression of Ki67 by comparing mean fluorescence intensity (MFI) (Figure 1). Our result shows that the Ki67 MFI of control HOVEC cells is 706±16. Interestingly, we found that MSC treated HOVEC cells shows significantly higher Ki67 MFI (1,194±96, P<0.05). This result indicates that the proliferation marker Ki67 expression level was increased more than 50% by MSC CM treatment.

### 4.2. MSC CM increases angiogenesis markers expression in HOVEC

**Figure 2:**
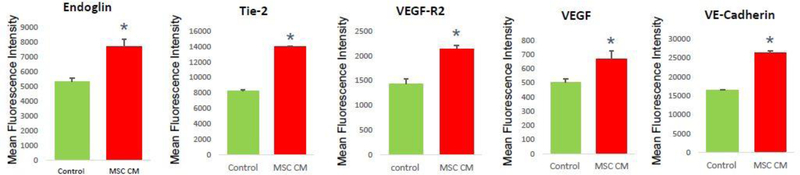
Angiogenesis marker analysis of HOVEC cells. HOVEC cells which were cultured with control media (control) and 50% MSC CM containing media (MSC CM) are stained with fluorescence conjugated anti-Endoglin, Tie-2, VEGF-R2, VEGF, and VE-Cadherin antibody and analyzed mean fluorescence intensity of positive population using FACS. (P<0.05).

Although we confirmed the enhanced proliferation of HOVEC by MSC CM, however, it is still unclear that MSC CM is increasing angiogenesis functions of HOVEC cells. To analyze actual angiogenesis, first, we analyzed the angiogenesis marker expression in HOVEC cells by FACS assay. We stained cells with various angiogenesis markers such as Endogline, Tie-2, VEGF-R2, VEGF, and VE-Cadherin. We compare marker expression level based on mean fluorescence intensity (MFI) which representing marker protein expression level in cells (Figure 2).

Interestingly, we found that in MSC CM treated HOVEC cells, the major angiogenesis marker VEGF and VEGFR was increased more than 30%. Other angiogenesis markers such as Endoglin, Tie-2, and VE-Cadherin also increased in MSC CM treated HOVEC cells. In our result, MFI of Endoglin in MSC CM treated HOVEC cells was approximately 40% higher compared to control HOVEC cells. The MFI of Tie-2 also increased by approximately 50% in MSC CM treated HOVEC cells.

MSC CM treated HOVEC cells also shows higher MFI in VEGF-R2, which is approximately 35% higher than control. In MSC CM treated group, the MFI of VE-Cadherin also 48% higher compared to the control group. The major angiogenesis factor VEGF also shows increased MFI in MSC CM treated group, which approximately 15% higher than control. With this result, we revealed that MSC CM can increase angiogenesis marker expression level.

### 4.3. MSC CM upregulate angiogenesis in HOVEC through PI3K/ALK pathway

**Figure 3:**
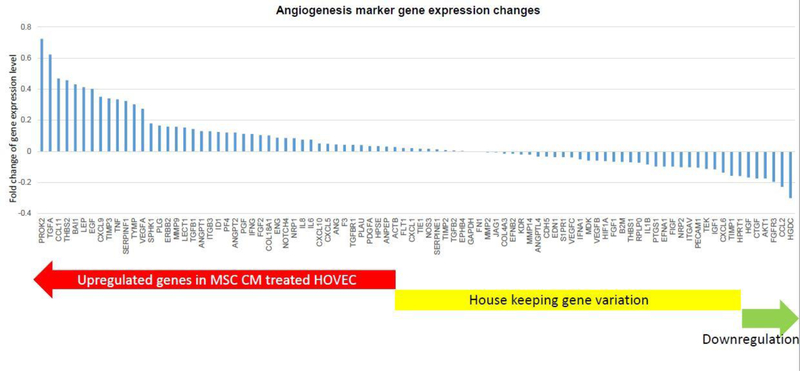
Angiogenesis marker expression changes in MSC CM treated HOVEC cells. mRNA were isolated from HOVEC cells which cultured with regular media (control) and MSC CM containingmedia. 84 key genes involving angiogenesis were analyzed using RT2 profiler PCR array. Gene expression level inMSC CM treated HOVEC were compared to control HOVEC cells.

Our results demonstrate that MSC CM can affect ovarian angiogenesis through stimulated HOVEC cells. As a next step, we want to know which pathway involved in this MSC CM related effect. There are a lot of well-known angiogenesis genes which probably regulated by MSC CM. We analyzed those angiogenesis gene expression changes in HOVEC cell by super array kit (Figure 3). We tested 84 key angiogenesis marker gene expression using RT2 profiler PCR array (QIAGEN). We found that half of these genes were expressed higher in MSC CM treated HOVEC cell. 42 angiogenesis marker genes such as TGFA, EGF, VEGFA, and ANGPT2 were upregulated in MSC CM treated HOVEC cells. Another 42 genes such as Flt1, TGFB2, and VEGFB were not significantly changed, and the other 5 genes were slightly down regulated compared to control HOVEC cells. We also figure out top 15 angiogenesis stimulator gene which shows the highest gene expression level after MSC CM treatment and investigate their function in angiogenesis through works of literature (Table 1). As a result, we found that among those highly expressed genes, most signaling molecules such as TGFA, CCL11, LEP, and EGF are involved in PI3K/ALK pathway. This result suggests that MSC CM stimulate angiogenesis and this stimulation mainly regulated by PI3K/ALK pathway.

### 4.4. MSC CM stimulate the tube forming ability of HOVEC

**Figure 4:**
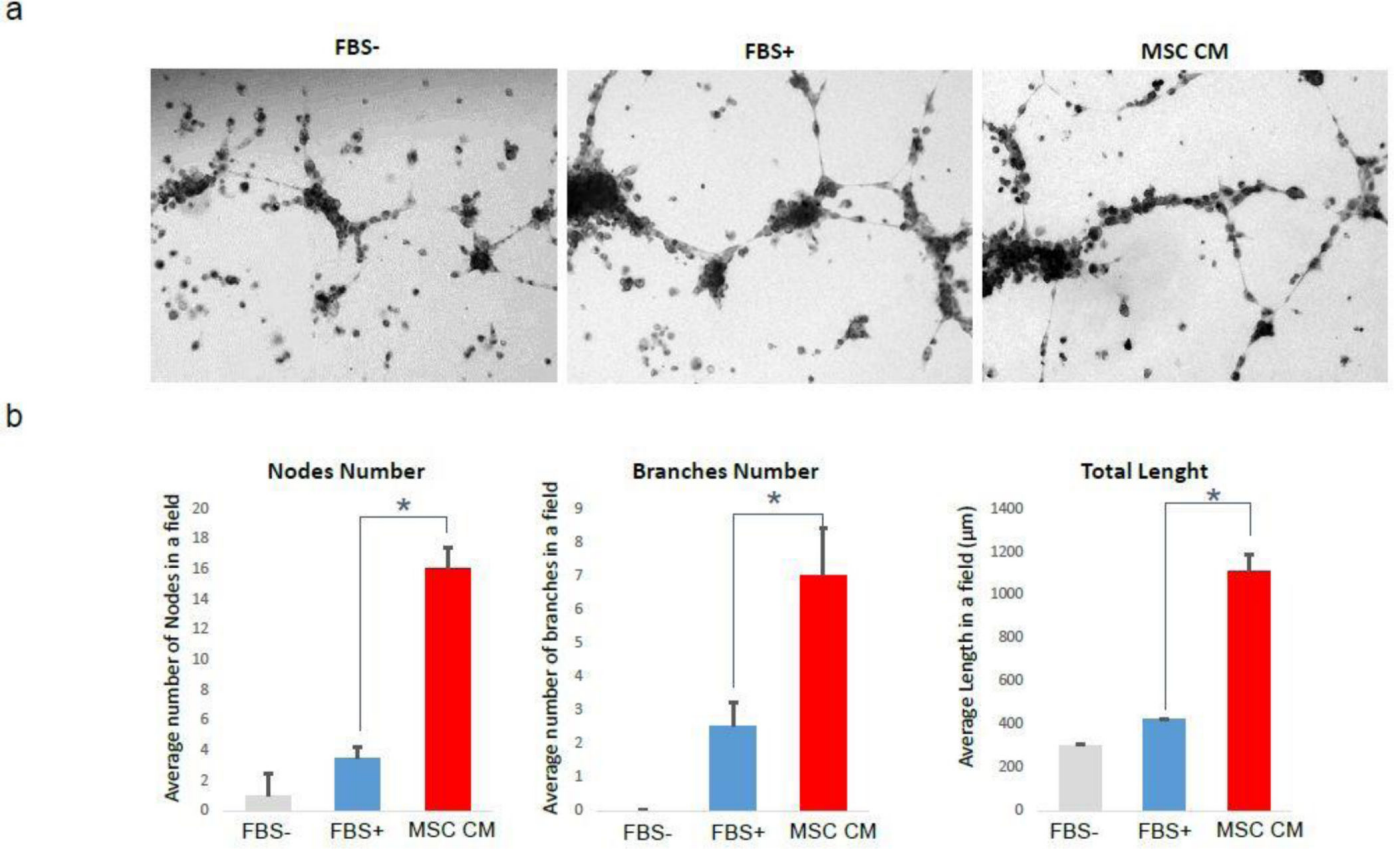
Tube formation analysis of HOVEC cells. HOVEC cells which were cultured on matrigel with regular media without FBS (FBS-), same mediaHOVEC cells which were cultured on matrigel with regular media without FBS (FBS-), same media with FBS (FBS+) and 50% MSC CM containing media (MSC CM). After 18 h of incubation, tube formation was analyzed by microscopy. (a) Bright field image of tube forming HOVEC cells on matrigel. (b) Quantitative comparison of number of nodes, branches, and length of tube in each group (P<0.05).

Endothelial cells have tube forming ability in vitro condition, and it representing angiogenesis by endothelial cells in vivo condition. In our results described above, it is clear that MSC CM can enhance the proliferation and angiogenesis marker expression in HOVEC cells. For the last step, we analyzed the actual tube formation ability of HOVEC cells, by tube forming assay using matrigel (Figure 4). We plated HOVEC cells and cultured without FBS (FBS-), with FBS (FBS+), and with 50% MSC CM (MSC CM) on matrigel coated 24-well culture plate. After 18 hours of incubation, cells forming tube formation and tubes are observed with EVOS microscope. We counted the number of nodes and found that average 1 node per field of view in the FBS- group. In contrast, cells in FBS+ group shows an average of 3.5 nods in the same field. Interestingly, cells in MSC CM group shows an average of 16 nods in the same field which is more than 4 fold higher compared to FBS induced tube forming condition. We also count the number of branches and compared it between groups. In FBS- group, we couldn’t find any significant branches. In FBS+ group, we found an average of 2.5 branches in each field of view. MSC CM group shows an average of 7 branches in the same field which is approximately 3 times higher than FBS induced tube forming condition. The length of the tube also shows a similar result. In FBS- group, length of total tube in each field of view was approximately 302 μm. In FBS+ group, length of total tube in the same field was 420 μm. interestingly; we found that length of tube in MSC CM group was 1108.5 μm, which are more than 2.5 fold higher than FBS induced tube forming condition. This result support that MSC CM treated HOVEC cells shows much higher tube formation ability.

## 5. Discussion

In chemotherapy-induced POI patients, reduced ovarian vascularization and blood vessel damage such as non-matured blood vessel formation, thickening and diffuse hyalinization of the wall are commonly reported in previous studies [5,20]. These damaged blood vessels lead to low viability of ovarian follicles and other ovarian cells. In this study, we propose MSC CM as a therapeutic component for chemotherapy-induced POI through enhancing ovarian angiogenesis. We reveal that MSC CM can stimulate human ovarian microvascular endothelial cells (HOVEC), increase angiogenesis marker gene expression in HOVEC, and stimulate tube formation. Furthermore, our PCR array study results demonstrate that MSC CM can stimulate angiogenesis in HOVAC cells via the PI3K/ALK pathway.

Ovarian angiogenesis capabilities of HOVEC cells can be promoted by increasing the number of HOVEC cells in the ovary. According to our hypothesis, MSC can promote HOVEC effect on angiogenesis. In the first step to demonstrate our hypothesis, we analyzed the effects of MSC CM on the proliferation of endothelial cells. According to our result, HOVEC treated with MSC CM shows a higher population of Ki67 positive cells. Ki67 is one of the commonly used proliferation markers expressed in proliferating cells [21–23]. We analyzed Ki67 positive cells by FAC. Results showed MSC CM inducing an increase in the proliferation of HOVEC cells approximately by 10%.

We analyzed several well-known angiogenesis markers such as Endoglin, Tie-2, VEGF-R2, VEGF and VE-cadherin. Endoglin can activate angiogenesis via Alk1 related signaling pathway which regulating SMAD 1/5/8 pathway [24,25]. Tie-2 is a receptor protein of Angiopoietin-1 and 2 which regulate angiogenesis [26–28]. VEGF-R2 and VEGFA is a major regulator of angiogenesis [29–31]. VE-Cadherin is one of adhesion molecule in endothelial cells and also known as angiogenesis regulation factor [32,33]. These markers were analyzed by Mean Fluorescence Intensity (MFI) which represents the marker expression level of each cell. We found that the MFI of each angiogenesis markers is slightly increased in MSC CM treated HOVEC. This result suggests that MSC CM can stimulate angiogenesis marker expression compared than normal culture condition. The increased blood vessel formation will enhance blood supply in POI ovary and will restore folliculogenesis and ovulation. This may explain how MSC effectively treated POI in our previous studies using a mouse model and patient.

In our PCR array result, we analyzed 84 key angiogenesis genes. Among those genes, 50 genes expression has increased by MSC CM treatment. The gene which was most highly expressed after MSC CM treatment was Prokineticin 2 (PROK2). The PROK2 gene has known as a stimulator of ovarian angiogenesis by activation of MAPK and AKT pathway [34,35]. The next highly expressed gene Transforming Growth Factor-Alpha (TGFA) which stimulate angiogenesis in various tissue such as carcinoma. TGFA signaling pathway also activates AKT via PI3K activation [36,37]. CCL11 which third-ranked highly expressed angiogenesis gene in MSC CM treated HOVEC cell is also enhanced angiogenesis via PI3K/AKT pathway [38]. When analyze only top 10 angiogenesis activator genes which were expressed higher in MSC CM treated HOVEC cell, 6 of 10 genes are involved in the PI3K/AKT pathway. These results suggest that the key mechanism of our MSC CM treatment that enhances angiogenesis is highly related to the PI3K/AKT pathway.

In our study, the stimulation effect of MSC CM on actual angiogenesis was confirmed by tube formation assay. It is reported that low serum condition inhibits tube formation and serum in culture media can stimulate tube formation by dose-dependent manner [39,40]. In tube formation assay study using endothelial cells, we investigated the effects of MSC CM on its tube formation ability. We used three different culture conditions depend on FBS existence. In our study, the first group of HOVEC did not expose in FBS during tube formation (FBS-). In this serum-free condition, a very small number of tubes were observed as we expected which representing the low angiogenic condition. The second group of HOVEC were exposed in 10% FBS during tube formation and shows regular tube forming ability (FBS+). The third group of HOVEC was cultured 1:1 mixed media of serum-free media and MSC CM (MSC CM). Due to our MSC CM also does not contain serum, HOVEC in MSC CM group also did not expose in serum during tube formation. In our result, we found that MSC CM treated group is significantly higher in tube formation, number of nodes, branches, and length of tubes compared to the other two groups. These results suggest that MSC CM stimulates angiogenesis 10% more than FBS.

Our findings also matched with previous studies for POI. In a published study using rat POI model, they found a decreased number of a blood vessel in chemotherapy-induced POI rat ovary and it recovered by adipose-derived MSC treatment [41]. The other study also shows that ovarian angiogenesis and VEGF expression can be decreased in chemotherapy-induced POI rat model and can be recovered by adipose-derived MSC through a paracrine mechanism [42]. These studies support our results and explain that ovarian angiogenesis can be stimulated by MSC CM through a paracrine effect on the ovarian endothelial cell.

In our study, we found the therapeutic effects of MSC CM to POI through angiogenesis. It seems to be clear that some factors in MSC CM can stimulate ovarian angiogenesis. However, it is still not clear which component of MSC CM is the key molecule in this effect. MSC CM containing various stuff such as metabolic products, Extracellular Vesicle (EV), miRNA and secreting proteins including cytokines. Among those components, miRNA in EV and protein are the strongest candidate of this therapeutic effect due to their paracrine effect. If we can identify the key molecule of POI treatment, we can isolate that molecule and inject into POI ovary as high dose to increase the therapeutic effect. We can also generate genetically engineered MSC as bio-organ which secret more factors for better therapeutic effect. This approach will be an interesting topic for our future study. Our study shows that MSC CM obtained from MSC induces proliferation of endothelial cells promoting Tube formation thus, enhances angiogenesis and increase the density of newly formed blood vessels. Further characterization of these factors can lead to novel therapeutic options for women with premature ovarian insufficiency and other causes of female infertility.

## Figures and Tables

**Table 1:** Top 15 angiogenesis stimulator genes expressed in MSC CM treated HOVEC.

Gene	Expression change	Function
PROK2	0.722876059	Ovarian angiogenesis via MAPK and PI3K/AKT pathway [34,35].
TGFA	0.62270244	angiogenesis in carcinoma via PI3K [36,37]
CCL11	0.467909147	angiogenesis via PI3K/AKT [38]
LEP	0.412112411	angiogenesis via JAK2/STAT3 and PI3K/AKT [43]
EGF	0.40089547	angiogenesis via PI3K/AKT [44,45]
CXCL9	0.35104549	angiogenesis via MAPK/APK [46]
TNF	0.333847649	angiogenesis via NF-KB [47,48]
TYMP	0.301952601	oxidation stress, increase VEGF [49]
VEGFA	0.272543655	Stimulate Angiogenesis
SPHK1	0.179885402	Activate S1P and AKT pathway [50]
PLG	0.16577538	Plasminogen, support VEGF, bFGF induced angiogenesis [51]
ERBB2	0.159172594	Stimulate VEGF expression [52]
MMP9	0.158525877	Key angiogenesis protein. Stimulated by PI3K/AKT pathway [53,54]
LECT1	0.153201634	Leukocyte Cell Derived Chemotaxin 1
TGFB1	0.142750411	Angiogenesis via Notch signaling pathway [55]
